# Insights on NIRS Sensitivity from a Cross-Linguistic Study on the Emergence of Phonological Grammar

**DOI:** 10.3389/fpsyg.2013.00170

**Published:** 2013-04-16

**Authors:** Yasuyo Minagawa-Kawai, Alejandrina Cristia, Bria Long, Inga Vendelin, Yoko Hakuno, Michel Dutat, Luca Filippin, Dominique Cabrol, Emmanuel Dupoux

**Affiliations:** ^1^Graduate School of Human Relations, Keio UniversityTokyo, Japan; ^2^Institut d’Etudes de la Cognition, Ecole Normale SupérieurParis, France; ^3^Neurobiology of Language, Max Planck Institute for PsycholinguisticsNijmegen, Netherlands; ^4^Department of Psychology, Harvard UniversityCambridge, MA, USA; ^5^Laboratoire de Sciences Cognitives et Psycholinguistique, EHESSENS-IES, CNRS, Paris, France; ^6^AP-HP Cochin Port RoyalParis, France

**Keywords:** near infrared spectroscopy, phonotactics, phoneme perception, infant, speech perception

## Abstract

Each language has a unique set of phonemic categories and phonotactic rules which determine permissible sound sequences in that language. Behavioral research demonstrates that one’s native language shapes the perception of both sound categories and sound sequences in adults, and neuroimaging results further indicate that the processing of native phonemes and phonotactics involves a left-dominant perisylvian brain network. Recent work using a novel technique, functional Near InfraRed Spectroscopy (NIRS), has suggested that a left-dominant network becomes evident toward the end of the first year of life as infants process phonemic contrasts. The present research project attempted to assess whether the same pattern would be seen for native phonotactics. We measured brain responses in Japanese- and French-learning infants to two contrasts: Abuna vs. Abna (a phonotactic contrast that is native in French, but not in Japanese) and Abuna vs. Abuuna (a vowel length contrast that is native in Japanese, but not in French). Results did not show a significant response to either contrast in either group, unlike both previous behavioral research on phonotactic processing and NIRS work on phonemic processing. To understand these null results, we performed similar NIRS experiments with Japanese adult participants. These data suggest that the infant null results arise from an interaction of multiple factors, involving the suitability of the experimental paradigm for NIRS measurements and stimulus perceptibility. We discuss the challenges facing this novel technique, particularly focusing on the optimal stimulus presentation which could yield strong enough hemodynamic responses when using the change detection paradigm.

## Introduction

When listening to speech, the human brain must process various aspects of auditory signals instantly over a series of levels: beginning with the acoustic and phonemic levels, through lexical access, syntactic integration, and up to the level of semantic interpretation. Infants begin to set the foundations of their language-specific knowledge that allows these computations well before they begin to talk (Kuhl, 2011). One of the early landmarks of language acquisition concerns learning the rules that govern sound sequences, or phonotactics, which differ in important ways across languages. For example, whereas English allows for two or more consonants at the beginning, middle, or end of the word, Japanese does not tolerate such consonant clusters. By the age of 9 months, infants show a preference for words that follow the phonotactics of their ambient language (Jusczyk et al., 1993), indicating that by this age they have begun to acquire their native phonological grammar. This phonotactic knowledge affects the formation of abstract sound categories (since infants decide whether two sounds map onto a single or two phonemic categories; White et al., 2008), the extraction of word forms from running speech (as illegal clusters are treated as word boundaries; Friederici and Wessels, 1993; Mattys and Jusczyk, 2001), and the acquisition of word form-meaning associations (because toddlers learn to associate meaning more easily to items with high-frequency phonotactics, compared to low-frequency ones; Graf Estes et al., 2011).

Another example of the impact of phonotactics on perception comes from perceptual repair, the process in which listeners report hearing a legal sequence of sounds even when they had been presented with an illegal sequence. For example, adult Japanese speakers tend to report hearing/abuna/when presented with/abna/, because consonant sequences (clusters) are illegal in Japanese (Dupoux et al., 1999). A recent behavioral study with infants has documented the developmental emergence of this effect (Mazuka et al., 2011): at 8 months of age, Japanese infants were able to discriminate between Abna-type and Abuna-type words, whereas by 14 months they had lost this ability. In contrast, French infants succeeded at both ages. These findings indicate that language-specific phonotactic constraints can affect perception even before infants have learned to speak, a timeline that coincides with the emergence of native perception for phonemic categories (e.g., Werker and Tees, 1984).

Studies with a variety of neuroimaging methods have only begun to reveal the neurophysiological underpinnings of the development of language networks in the infant brain (Minagawa-Kawai et al., 2008; Gervain et al., 2010; Kuhl, 2011). A particularly fruitful avenue of research combines a change detection paradigm and a hemodynamically based, child-friendly method called Near InfraRed Spectroscopy (NIRS). In *baseline* blocks, infants are presented with a repeated background stimulus (e.g., itta itta itta …). In *target* blocks, infants are presented with alternating items (e.g., itta itte itta itte …). The contrast between baseline and target blocks is thought to reveal the areas engaged in the discrimination of the two types presented during target blocks. Research using this method reveals that brain activation to phonemic categories becomes left-lateralized toward the end of the first year (e.g., Sato et al., 2003, 2010; Minagawa-Kawai et al., 2007; a recent summary in Minagawa-Kawai et al., 2011a). However, there is no data on the neural network subserving infants’ processing of native phonotactics.

In fact, adult fMRI research suggests that there is a considerable overlap between the network recruited for native phonotactics and that involved in native phonemic processing. Jacquemot et al. (2003) presented Japanese and French adults with pseudo-word triplets, which were drawn from three possible types:/abna/(containing a cluster),/abuna/(containing a short vowel), and/abuuna/(containing a long vowel). Some trials contained identical triplets, others contained a contrast between cluster and short vowel, and yet others contained a contrast between short and long vowel. Participants’ task was to decide whether or not the last item in the triplet was physically identical to the preceding two. Notice that the duration contrast contained a phonological change for Japanese listeners but not for French adults, whereas the converse was true for the cluster contrast. Results showed that the phonological contrast in one’s native language activated the left superior temporal gyrus (STG) and left supra marginal gyrus (SMG) to a greater extent than the non-phonological contrast for both French participants (i.e., /abna, abuna/ > /abuna, abuuna/) and Japanese participants (i.e., /abuna, abuuna/ > /abna, abuna/). Activation in left STG (including the planum temporale) was interpreted to reflect phonological processing, while SMG activation appeared to be related to the task’s loading on phonological short-term memory. Notice that, despite tapping phonotactic and phonemic knowledge respectively, the two contrasts activated a similar cerebral network (see also Friedrich and Friederici, 2005; Rossi et al., 2011, for lexical tasks tapping phonotactic knowledge).

In summary, a wealth of behavioral research has shown that both phonotactic and phonemic knowledge emerge toward the end of the first year. Moreover, contrasts between native sound categories come to involve a left-dominant brain network around this age as well. Finally, adult neuroimaging work suggests that there is an overlap between the network processing phonemic and phonotactic dimensions, although the crucial data on this is missing in infancy. The present study thus set out to complete this picture. We used a change detection paradigm similar to those previously used in NIRS research to study the brain network involved in processing two types of contrasts: one relying on phonotactic knowledge and the other on phonemic sound categories. The present investigation is to our knowledge the first cross-linguistic NIRS study, as we tested both Japanese and French infants on their perception of clusters and vowel duration contrasts. In spite of being theoretically well-motivated, however, we forewarn readers that we found very weak evidence of change detection for either contrast or population. Although in the following section we show the cerebral response data to phonological grammar in two different language groups, this paper will mainly discuss the factors likely led to these null results. We are certain that these null results are not due to simple low-level factors (such as a malfunctioning NIRS machine or low stimuli quality), as we assured that our NIRS machines successfully captured Hb responses in the infant brain in our previous work with the same probe pads and basic NIRS paradigm (Japan: Minagawa-Kawai et al., 2007, 2011c, France: Minagawa-Kawai et al., 2011c; Cristia et al., 2013). Other variables considered were: the acoustic salience of the contrasts when embedded in a word, participants, the design of the NIRS probe pads, and the particular experimental paradigm. After careful reflection, we reasoned that one methodological parameter of the stimulus presentation was the most likely cause, and therefore conducted an additional adult NIRS experiment to directly examine this factor. Together with these additional data, we discuss the optimal method to evoke strong enough Hemoglobin (Hb) responses while taking into account the relative limitations of this novel technique. As previous studies have rarely reported null results, the present report will contribute to more efficient experiment planning for infant NIRS studies.

## Materials and Methods

### Participants

There is considerable variability in the exact timeline of the emergence of language-specific effects, sometimes reported as early as 6–8 months or as late as 27 months (a review in Tsuji and Cristia, submitted). Moreover, this emergence is not always stable. For example, discrimination responses to a duration vowel contrast showed W-shaped changes in infants across 3- to 14-months-old (Minagawa-Kawai et al., 2007). Therefore, we tested Japanese infants at a wide range of ages, from 3 to 14 months, in order to explore the stability of neural bases of attunement to the phonological grammar. We also made age groups similar to those of Minagawa-Kawai et al. (2007). Specifically, the following numbers of infants were included in the analyses: 15 within the group of 3–5 months [3–5 m] (9 males; *M* = 4:5; range 3:4–5:12); 11 6–7 m (8 males; *M* = 7:3; range 6:5–7:28); 15 8–9 m (8 males; *M* = 9:1; range 8:4–9:30); 15 10–11 m (12 males; *M* = 10:29; range 10:1–11:22); 10 12–14 m (6 males; *M* = 13:16; range 12:8–14:27). Thus, to examine the developmental change of neural response to native and non-native phonotactic contrasts, we focused on Japanese infants by measuring them at various ages from 3- to 14-months-old. Furthermore, a previous behavioral study using similar phonotactic stimuli for Japanese and French infants reported the language-specific difference at the age of 14-months-old. Therefore we also tested 14-months-old French infants to contrast to Japanese infants at the same age. Twenty 14-month-olds were included (12 males; age *M* = 14:3 range 13:19–14:14). All participants had been born full-term being raised in a largely monolingual home, with no exposure to the other language under analysis here (i.e., no Japanese exposure in French infants; no French exposure in Japanese infants). Japanese infants were tested in Tokyo, and French infants in Paris. In addition, 11 French and 35 Japanese infants participated but their data were excluded from analysis for the following reasons: not enough data (i.e., less than four good trials in each condition, *N* = 29), cried or were fussy (*N* = 10), exposure to the other language being tested here (*N* = 2), and technical error (*N* = 5). Consent forms were obtained from parents before the infants’ participation. This study was approved in Japan by the ethic committee of Keio University, Faculty of Letters (No. 09049); and in France through the Ile de France III Ethics Committee (No. ID RCB (AFSSAPS) 2007-A01142-51).

### Stimuli

Three types of non-words/abna/,/abuna/and/abuuna/were used as stimulus words. Three tokens of each word (i.e., a total of nine tokens) were chosen from recordings made by a female bilingual speaker of Japanese and French so that the vowels and consonants in the stimuli were good tokens of the category in both languages. These tokens were clearly pronounced in an infant-directed speech fashion and their acoustic details are shown on Table 1. As shown in the Table, we selected tokens for/abna/that had no vowel-like waveform between the two consonants. The present procedure used three exemplars for each word to make the phonetic variability higher and thus closer to that of a natural context (Mazuka et al., 2011). The word/abuna/was used as a baseline stimulus, and the other words served as two target conditions as contrast to the baseline: cluster (/abna/) and vowel duration (/abuuna/) conditions. Following the general change detection paradigm widely used in NIRS studies (e.g., Furuya and Mori, 2003; Sato et al., 2003, 2010), we did not use silence period as a baseline. Instead we presented/abuna/between the target blocks. Thus in the stimulus presentation, a baseline block (9–18 s) and a target block (9 s) are alternated so that we could measure Hb change during the target block in contrast to the baseline block. To exclude the systemic vascular effects from the Hb signal, the duration of the baseline block was jittered, while the length of the target block was fixed as in a typical block design paradigm. Participants first heard a baseline block consisting of three variations of/abuna/for 9–18 s with a stimulus onset asynchrony (SOA) of 1.5 s; thus, one baseline block contains 6–12/abuna/tokens. In a cluster target block,/abna/and/abuna/were pseudo-randomly presented for 9 s with the same SOA. Similarly, in a vowel lengthening target block,/abuuna/and/abuna/were pseudo-randomly presented. Baseline blocks (9–18 s) and the target blocks were alternated for at least 16 times (8 times per condition) and a maximum of 30 times. This resulted in a total duration with a minimum of about 6.5 min to a maximum of 11.5 min. The order of the two different target conditions was also presented pseudo-randomly.

**Table 1:** **Acoustic information of the stimuli**.

Duration (ms)	Abuna					Abna				Abuuna				
	/a/	/b/	/u/	/n/	/a/	/a/	/b/	/n/	/a/	/a/	/b/	/u:/	/n/	/a/
Phoneme	115.4	88.8	128.2	99.9	151.8	112.1	127.6	78.1	140.8	126.3	88.8	369.1	88.3	150.0
SD	2.5	23.4	27.9	4.4	5.3	4.3	3.0	2.6	2.6	5.7	18.5	25.3	10.3	6.2
Word	584.0 (70.2)					458.6 (7.9)				822.4 (18.0)				
Pitch (Hz)	Minimum		187.2 (10.7)			Minimum		190.3 (5.5)		Minimum		191.3 (1.5)		
Range	max		239.3 (20.1)			max		243.0 (2.6)		max		241.3 (1.5)		
Average	213.6 (10.5)					225.3 (14.0)				221.0 (2.0)				

*Averaged duration of phonemes and words, pitch range and averaged pitch values for each word type are shown. Values inside parenthesis are standard deviation. No accentuation is assigned to these stimuli*.

### Procedures

**Figure 1:**
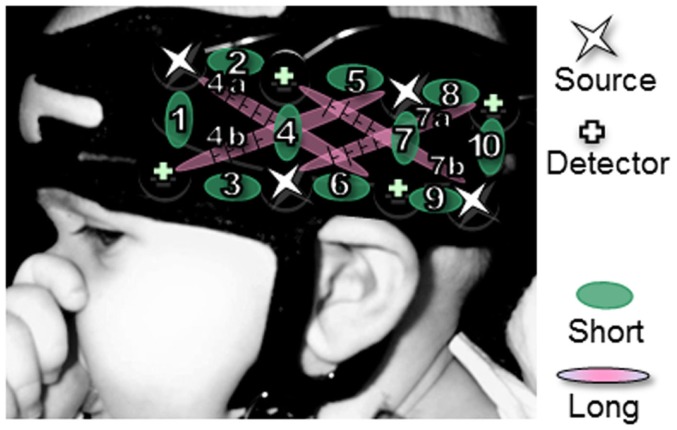
**Pad placement**. Location of the pads relative to surface landmarks on a typical 5-month-old infant’s head. Channels with a long distance (4a,b,7a,b) are only applicable to the French results.

Infants were tested either in Paris or Tokyo. The actual NIRS machines differed across the two labs (Paris: UCL-NTS, Department of Medical Physics and Bioengineering, UCL, London, UK; Tokyo: ETG-7000, Hitachi Medical Co., Japan) (Everdell et al., 2005). Both systems provide estimates of Hb concentration changes of the optical paths in the brain between the nearest pairs of incident and detection optodes. Both systems emit two wavelengths (approximately 780 and 830 nm for ETG-7000, 670 and 850 nm for UCL-NTS) of continuous near infrared lasers, modulated at different frequencies depending on the channels and the wavelengths, and detected with the sharp frequency filters of lock-in amplifiers (Watanabe et al., 1996). The same probe geometry was used in both labs, which is represented in Figure 1. There was one pad over each temporal area, which was placed using anatomical landmarks to align the bottom of the pad with the T3-T5 line in the international 10–20 system (Jasper, 1958). Each pad contained four emission and four detection optodes, arranged in a 2 × 4 rectangular lattice. These optodes were placed in their respective temporal areas with a source-detector separation length of 25 mm (Watanabe et al., 1996; Yamashita et al., 1996). This separation enables us to measure hemodynamic changes up to 2.5–3 cm deep from the head surface, which traverses the gray matter on the outer surface of the brain (Fukui et al., 2003). Given this geometry, measurements from each hemisphere can be derived from 10 channels between adjacent sources and detectors, and – only in the UCL system used in Paris – 4 channels between non-adjacent sources and detectors, at a distance of 56 mm. For ease of expression, we will call the former channels “short-distance” (since they are defined by two optodes at a shorter separation) and the latter “long distance.”

Once the cap was fit on an infant participant, the stimuli were presented from a loudspeaker. The stimulus sounds played by a PC were presented at an amplitude of about 70 dB SPL, measured at the approximate location of the infant’s head sitting on a caregiver’s lap in the center of a sound-attenuated booth. To reduce motion artifacts, an experimenter entertained the infant with silent toys during the recording.

### Data analysis

#### Artifact detection, baselines, and detrending

Intensity signals were converted into oxygenated hemoglobin (oxy-Hb) and deoxygenated (deoxy-Hb) hemoglobin concentration using the modified Beer-Lambert Law. The state-of-the-art methods in infant NIRS analyses profit from insights that have been gained in more established hemodynamic methods, including the use of General Linear Models (GLM; for example, GLM was applied in the infant studies reported in Telkemeyer et al., 2009; Kotilahti et al., 2010; Minagawa-Kawai et al., 2011a,b). In such state-of-the-art analyses, artifacts are assessed at the level of probes (rather than channels; see e.g., Kotilahti et al., 2010, for a discussion of advantages); and by using the criterion of changes larger than 1.5 mM/mm (millimolars per millimeter) within 100 ms in band-passed (0.02–0.7) filtered total hemoglobin (Pena et al., 2003; Gervain et al., 2008; see Minagawa-Kawai et al., 2011a for discussion). Artifacted stretches of the signal are excluded from the analyses by giving them a weight of zero in the GLM. Additional regressors accounted for baseline changes following major artifacts (through boxcars), and slow non-linear trends (sine and cosine regressors with periods of 2, 3, … *n* min, up to the duration of the session).

Activation levels were estimated by assessing the correlation between the signal observed and the signal predicted by convolving the canonical hemodynamic response with boxcars for two experimental regressors, one for each of the two conditions (Cluster, Duration). Individual channels were judged as responding if their degree of correlation was higher than expected by chance, using Monte Carlo bootstrap resampling to correct for multiple comparisons (Westfall and Young, 1993; *N* = 10,000). Planned analyses involved entering the beta values from responding channels (and their hemispheric counterparts) into an Analyses of Variance (ANOVA) to assess effects of Condition (Cluster, Duration), Hemisphere (Left, Right), and their interactions, for different age groups. Such an analysis has been used to document the emergence of left-dominant responses to sound changes, and right-dominance to prosodic changes, in Sato et al. (2010). Applying the same analysis to the current study, we predicted that Japanese infants at 12–14 months of age would exhibit greater responses in left channels for the Duration versus the Cluster blocks, whereas both Duration and Cluster blocks would lead to largely bilateral responses in younger Japanese children. In contrast, the French 14-month-olds should exhibit greater responses in left than right cortices for Cluster blocks, but not Duration blocks.

Another way of measuring lateralization involves the calculation of laterality indices, estimated as the difference in activation in left compared to right channels, divided by the total activation. Laterality indices have most frequently been estimated in previous infant NIRS work using the maximum absolute total-Hb observed within a range of channels defined as a Region of Interest (ROI) due to their likelihood of tapping auditory cortices (Furuya and Mori, 2003; Sato et al., 2003; Minagawa-Kawai et al., 2007). To calculate laterality indices, we reconstructed the hemodynamic response function for each infant, channel, and condition by fitting a linear model with 20 one-second boxcar regressors time-shifted by 0, 1, …, 20 s respectively from the onset of the target block. We then extracted the value of the maximum absolute total-Hb among the resulting betas for 0–9s within channels 4, 6, and 7 (in the left and right hemisphere), and inputted these values into the formula [L-R]/[L + R]. As with the ANOVA analyses, we expected laterality indices to be significantly above zero only for the Duration blocks in Japanese 12- to 14-month-olds, and for Cluster blocks in French 14-month-olds.

## Results

**Figure 2:**
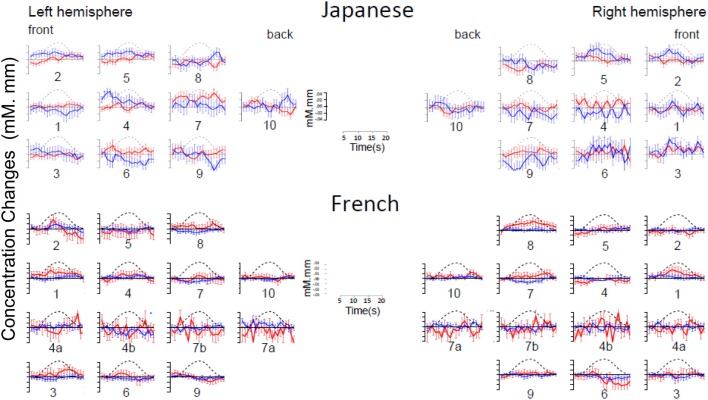
**Time course of hemoglobin responses**. The top panel shows the time courses of oxy-Hb (red) and deoxy-Hb (blue) in the 10 left and 10 right channels recorded in the Japanese infants (collapsing across all ages, *N* = 66). The bottom panel shows the same in the 14 left and 14 right channels recorded in the French infants (*N* = 20). For both panels, we have collapsed across conditions for ease of inspection (see Figures A1 and A2 in Appendix for time courses separating by condition).

**Figure 3:**
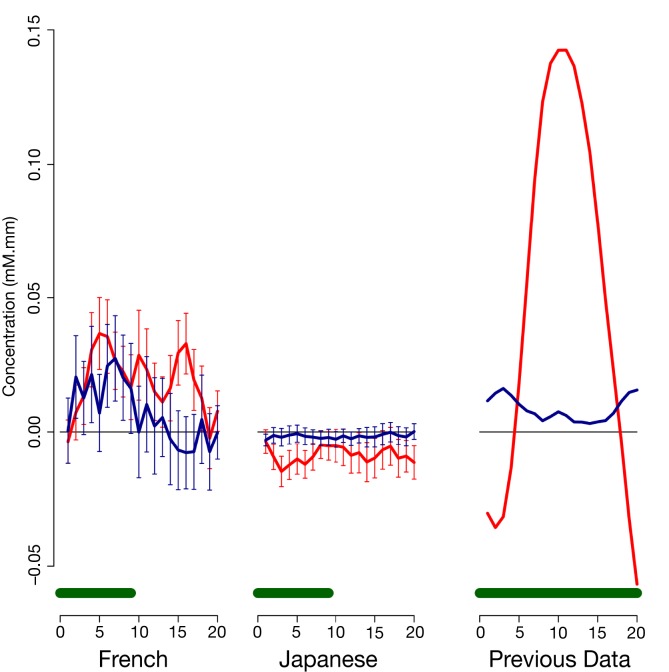
**Comparison of average time courses of hemoglobin responses with a previous study**. Each panel shows average and standard error for oxy-Hb (red) and deoxy-Hb (blue) within an auditory Region of Interest; the green bar at the bottom indicates the stimulation period. The left panel shows responses to duration block in Japanese infants in the present study (collapsing across ages); the middle panel responses to cluster blocks in French infants in the present study; and the right panel responses to a vowel quality change reported in previous research (Minagawa-Kawai et al., 2009).

As evident in Figure 2, none of the 10 channels measured from Japanese infants (top panel) or the 14 channels measured from French infants (bottom panel) responded significantly to the stimuli. Inspection of these waveforms clearly shows that the low ß were not due to infants’ responses deviating from adults’ responses along documented dimensions of variation (such as the width of the response or the extent of the subsequent undershoot; e.g., Handwerker et al., 2004), but rather because of an overall lack of response. That is, when observing individual channels and infants, it was not the case that oxy-Hb levels increased after the onset of the target block while deoxy-Hb decreased during that time. Instead, levels increased or decreased in both oxy-Hb and deoxy-Hb in tandem, or (most frequently) increased and decreased more or less randomly. For reference, Figure 3 compares the average HRF recovered from the current Japanese and French data with the average HRF recovered from another study (Minagawa-Kawai et al., 2009) using essentially identical equipment and procedure in our respective labs. Clearly, the HRF responses measured in the other studies were much more pronounced than those recorded in the current study.

**Figure 4:**
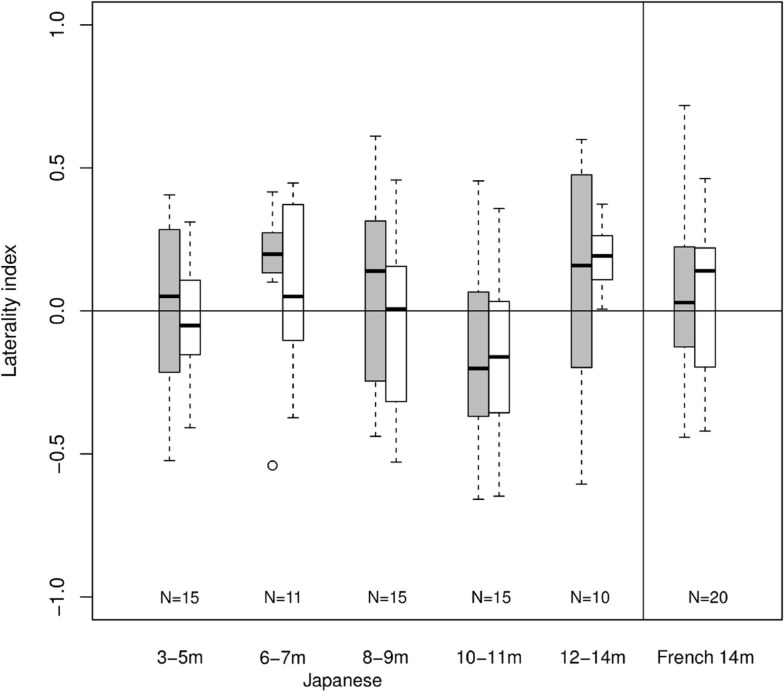
**Laterality indices**. Each bar indicates mean (black line), first and third quartile (edges of the box), and range (edges of the whiskers) in laterality indices within each age group and condition (gray for cluster, white for duration; *N* shown at the bottom).

Given the low overall responding level, no channels could be included in ANOVAs. Although the laterality index calculations remain theoretically possible, any departure from zero would be rather surprising given the lack of clear hemodynamic response. Figure 4 shows laterality indices by age group using maximum absolute total-Hb in the calculation. In this Figure, there is a trend for left-dominant activations for 6- to 7-month-old Japanese infants in response to Cluster blocks. A trend for left-dominance appears at 12- to 14-month-olds for Duration. The data from French infants does little to clarify the picture. There are no asymmetrical responses to any of Cluster and Duration blocks. Thus, laterality indices using maximum total-Hb lead to unreliable results in the present study, likely due to the lack of a clear hemodynamic response to the stimuli being tested.

## Discussion

**Figure A1:**
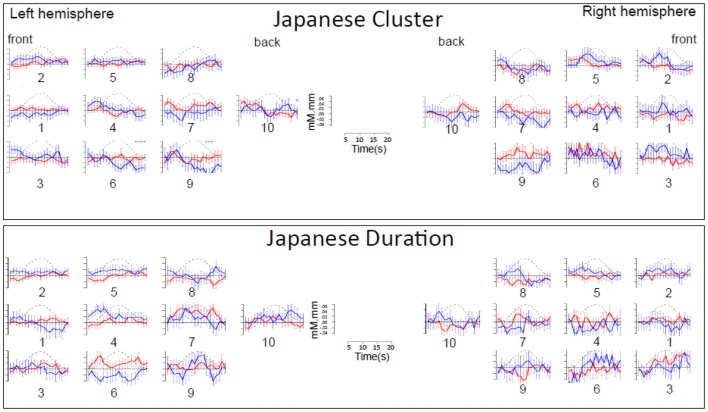
**Time course of hemoglobin responses by condition in Japanese infants (*N* = 66)**. The top panel shows the time courses of oxy-Hb (red) and deoxy-Hb (blue) in the 10 left and 10 right channels recorded in the Japanese infants (collapsing across all ages) during Cluster blocks. The bottom panel shows the same for Duration blocks.

The present study was the first to breach the question of the emergence of language-specific responses to phonotactic regularities. In this quest, we adopted a standard paradigm with some minor modifications, and used the same equipment and setup as in previous studies focusing on sound contrasts. Unlike previous studies, no response was apparent to contrasts in vowel duration, or contrasts between a bisyllable with a cluster versus a trisyllable. Furthermore, laterality indices were unreliable and variable, likely due to weak and variable Hb values, with no clear evidence for stable bilaterality early on, eventually replaced by left-dominance in conditions that were language-specific for the infant listeners. Although older Japanese infants showed left-dominant activations to the native Japanese vowel duration contrast in accordance with previous results (Minagawa-Kawai et al., 2007), this result may not be reliable as we did not have a clear Hb response to this contrast (Figure A1 in Appendix). In the remainder of this Discussion, we raise potential explanations for this null result, and argue that the most likely one takes into account both the low perceptibility of the phonotactic contrasts and the paradigm which is likely suboptimally suited to measure small hemodynamic responses.

Two possible explanations can be ruled out as unconvincing, namely insufficient sample size and inaccurate probe placement. Both in comparison with the general body of previous NIRS work and with published studies using the same method adopted here, sufficiently large numbers of infants were included. A recent systematic review of published infant NIRS research shows that the median number of infants per group in infant NIRS research is 15 (Cristia et al., 2013). More relevant to the current study, Sato et al. (2003) included between six and seven infants in each age group, and the smallest sample sizes per age group included in Minagawa-Kawai et al. (2007) was eight (at 25–28 months; sample sizes were larger for younger age groups: 3–4 months *N* = 15, 6–7 months *N* = 14, 10–11 months *N* = 11; at 13–15 months 2*N* = 9). In the present study, the smallest sample size was 10, with as many as 20 infants being included (French 14-month-olds). Pad location is also unlikely to have been a contributing factor, since we have previously been able to register responses using these precise pad locations to a variety of auditory stimuli in previous studies (Minagawa-Kawai et al., 2007, 2009, 2011a,b; Arimitsu et al., 2011). Indeed, while the Paris setup allowed for an even denser sampling through the use of multiple interoptode distances, no clear response to either change was evident in the French data.

One salient difference between the present study and previous NIRS work concerns the position of the contrast under study within words. All previous NIRS work on infants’ sound discrimination has made use of bisyllables, with the relevant contrast occurring in the final syllable (Sato et al., 2003; Minagawa-Kawai et al., 2007; Arimitsu et al., 2011). Our interest was in phonotactics; since word-medial position is where Japanese and French differ the most in terms of the sequences that are tolerated, we embedded both relevant contrasts in a middle syllable. However, some behavioral research in both toddlers (Nazzi and Bertoncini, 2009) and adults (Endress and Mehler, 2010) suggests that the perception of word edges is more accurate than the perception of word middles. By embedding the relevant contrasts in non-salient positions, we might have rendered the task more difficult for infants.

A possible argument against this explanation is that similar stimuli successfully elicited cross-linguistic differences in phonotactic perception patterns using behavioral methods (Mazuka et al., 2011). However, the infants in Mazuka et al. (2011) were actively attending to the sounds in order to control their presentation, whereas in the present study infants were being distracted with silent toys. It is well-known that even if a change is automatically detected, attention can greatly modulate the size of the response (Imaizumi et al., 1998). Although both ERP and fMRI have been effective in detecting cross-linguistic differences in adults of the precise type used here (Dehaene-Lambertz et al., 2000; Jacquemot et al., 2003), participants in those studies were also actively listening to and performing a task with the stimuli. However, distraction alone cannot account for the null result, given that the same procedure has been used in all previous infant NIRS studies that focused on the processing of native sound categories.

Two additional explanations likely played a key role in preventing the reliable measurement of Hb responses, namely insufficient block duration and token variability. Longer stimulation periods (the median is 15 s) are preferred in NIRS experiments as they are thought to increase the likelihood that increases in Hb concentration will accumulate to a point measurable beyond noise. Although event-related paradigms can in principle be used with NIRS (and fMRI), they are extremely rare, making up less than 10% of published infant NIRS studies (Cristia et al., 2013). This problem could be aggravated when using an oddball paradigm, like the one used in the present study, since the baseline period is not defined as the absence of stimulation but only the absence of change. Although one previous infant study has used block durations similar to the ones employed in the present study (Sato et al., 2010, 10 s), their SOA was 1 s, resulting in the presentation of 10 words versus 6 words (SOA = 1.5 s in 9 s block) in our study. Thus, the presentation of stimuli utilized in the present study may have contributed to the weaker response. The second explanation concerns the use of multiple tokens per stimulus word. Traditionally, both behavioral and neuroimaging studies that have used an oddball paradigm to study speech perception have used a single token per category. That is, a single sound recording is presented as the background stimulus, which is repeated over and over. This facilitates the construction of the auditory memory trace as participants can easily process and encode the precise acoustic representation of a single token. As a result, the contrast with between the background stimuli and the single token that represents the oddball becomes more salient. However, since the previous behavioral study showed that Japanese 14-month-olds could detect a vowel deletion change (cluster) with the use of a single token, we chose to use multiple tokens. Unfortunately, this may have ultimately weakened the automatic change detection response measured in our study. In order to examine the validity of these two explanations, we performed an additional NIRS experiment with Japanese adult participants.

Eight Japanese adult participants (five female; averaged age 35.6) were tested with the Hitachi system. In this study, we varied the procedure in order to investigate two methodological factors that could lead to weak signal-to-noise ratios: (A) the duration of stimulus block, and (B) token variability. Specifically, we compared two target block durations, 9 s (as in the current infant studies) and 15 s (which could allow further response build up). We also compared multiple tokens per stimulus word (as in the current infant studies) against a single token of each word (which should facilitate the discrimination task). Thus, we used the same stimulus and presentation of block design as employed in the infant study, but we manipulated target duration and token variability (in a 2 × 2 factorial design) to gather four sessions in each participant, counterbalanced in order. In these four sessions, the stimuli in the target block were (1) single tokens for 9 s (short-single), (2) single tokens for 15 s (long-single), (3) multiple tokens for 9 s (short-multi) and (4) multiple tokens for 15 s (long-multi). For the baseline block, we applied the same criteria of token variability used in the relevant target block (i.e., single tokens for 1 and 2, multiple tokens for 3 and 4); and the durations were jittered within 9–18 s for the short conditions (1 and 3) and 15–22 s for the long conditions (2 and 4). Each target block was presented 4–5 times, for a total session duration of 4–5 min. The experimental procedure differed from that of infants in the following ways: (1) adult participants paid attention to the stimuli without any distractions (e.g., toys); and (2) the distance between the optical probes was increased to 30 mm to take into account the difference in scalp and skull thickness of adults. Because our aim was to compare the response amplitude between the four conditions, we focused on the maximum Hb change from four channels corresponding to the vicinity of auditory regions, which typically show auditory evoked responses (Minagawa-Kawai et al., 2008). Furthermore, we only analyzed the data for the duration target blocks, containing the change “abuna-abuuna,” since the cluster target blocks should not elicit any strong responses due to the lack of consonant clusters in Japanese phonology.

**Figure 5:**
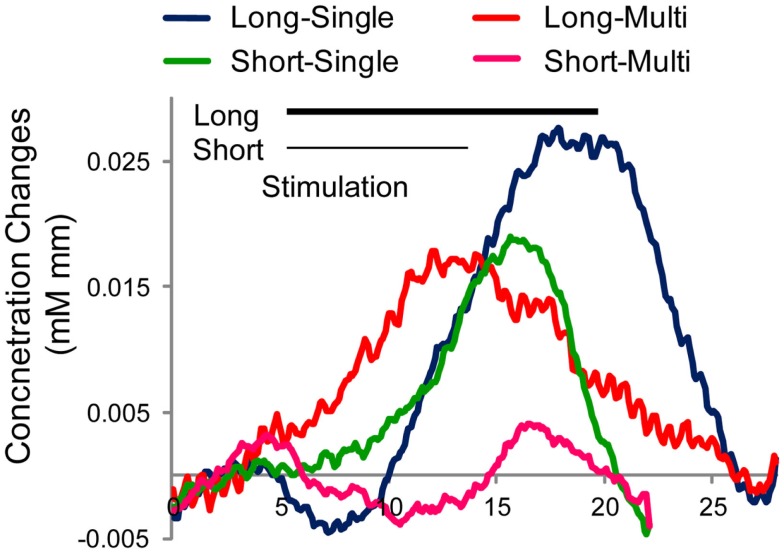
**Averaged time course of hemoglobin (oxy-Hb) responses around left auditory area by condition in Japanese adults (*N* = 8)**. Four stimulus conditions differ in target duration (Long or Short) and token variation (Single or Multiple tokens). Stimulation period is indicated by black line for the long stimulation (15 s) and gray line for the short stimulation (9 s).

The grand average of Hb time course is indicated in Figure 5. Clearly, the “long-single” condition elicited the largest and clear response among the four conditions. In contrast, “long-multi” and “short-single” evoked a weaker Hb response, with the weakest activation for “short-multi,” precisely the combination we implemented with infants. To confirm this tendency in Figure 5, an ANOVA with duration and token variability as two within subject factors was performed using *Z*-scores obtained from the GLM analysis. Results support the tendencies observed, showing main effects of these two factors [duration *F*(1,31) = 5.84, *p* = 0.046; token variability *F*(1,31) = 18.67, *p* = 0.004]^1^. These results confirmed our predictions that shorter target block duration and greater token variability negatively affect the amplitude of the evoked Hb response, which could constitute key factors in the infant study reported above. Moreover, the adult participants were paying attention to the stimuli, whereas infants were being distracted with silent toys, a factor that, as noted, may have further decreased the evoked responses.

Therefore, we can conclude that the present null results probably result from an interaction of multiple factors with overly short block duration and overly large token variability playing a main role, in addition to the other factors noted above (long SOA leading to few tokens in the target block, attention being drawn away from the stimuli, low salience of the change when embedded in trisyllabic words). Overall, these infant and adult results delineate some limitations of NIRS as a technique to measure infant perception. It has previously been pointed out that less repetition is required for NIRS measurements, and that this should be one of the advantages of NIRS over electro-encephalography (EEG) (Imaizumi et al., 1998; Furuya and Mori, 2003; Minagawa-Kawai et al., 2008). This intuition emerges from the fact that NIRS relies on a vascular response, which should be relatively stronger and more stable than a fast, event-related electrophysiological response. However, it would behoove NIRS researchers not be overly optimistic, and to bear in mind that the physical saliency of the stimuli, the choice of the experimental paradigm, and the stimulus presentation parameters are in fact crucially important and must be carefully selected to allow the vascular response to occur and the event-related Hb response to build up. This is particularly important for infant studies, where stable attention to the stimuli is less likely realistic.

This series of studies with both infants and adults have revealed a vulnerability of the change detection paradigm, frequently employed in the NIRS literature (Furuya and Mori, 2003; Sato et al., 2003, 2010; Minagawa-Kawai et al., 2007, 2011a). This paradigm has been widely used to assess the discrimination of phonemic and prosodic categories in both adults and infants (for a review, see Minagawa-Kawai et al., 2011a). Both previous adult fMRI work (Jacquemot et al., 2003) and infant behavioral research (Mazuka et al., 2011) that focused on the processing of the same kinds of contrasts studied here employed multiple tokens as stimuli. However, as we confirmed in a separate study, using multiple tokens in the NIRS-based change detection paradigm reduced the Hb signals. Thus, when all the evidence is taken into account, it appears that the change detection paradigm implemented in NIRS is a less sensitive index of discrimination abilities in infants than behavioral measures. While token variability reduced the observed NIRS responses, it did not prevent young Japanese infants or French toddlers from discriminating the exact same kinds of contrasts (Mazuka et al., 2011). Our follow-up adult study suggested that this noxious effect did not completely eliminate Hb responses, as indicated by weak but reliable response to the multiple stimuli condition in adults. This further suggests a significant role for attention during the change detection procedure. As a final remark, it should be pointed out that the change detection procedure which presents alternating and non-alternating stimuli is still a robust paradigm for various types of stimuli in NIRS studies (Minagawa-Kawai et al., 2011a). What we suggest here is that there is an optimal method to elicit strong Hb responses. We hope that this knowledge may strengthen future infant studies using NIRS.

## Conclusion

In summary, the present study sought to shed light on how the infant brain comes to code native phonotactics, and compare the resulting network with that found for native sound categories. Unfortunately, we were unable to observe a discrimination response for either phonotactics or a duration contrast that had been used in previous NIRS research. We have argued that the most likely explanations for the null result relate to an unfortunate combination of short target blocks, low stimulus perceptibility due to the use of multiple tokens and target position in the stimulus words, and low signal-to-noise ratio due to the lack of a task involving the stimuli. Future work may be wise to avoid such an outcome by carefully choosing the experimental parameters to obtain a strong enough hemodynamic response by varying stimulus saliency and/or directing infants’ attention to the stimuli.

## Conflict of Interest Statement

The authors declare that the research was conducted in the absence of any commercial or financial relationships that could be construed as a potential conflict of interest.

## Figures and Tables

**Figure A2:**
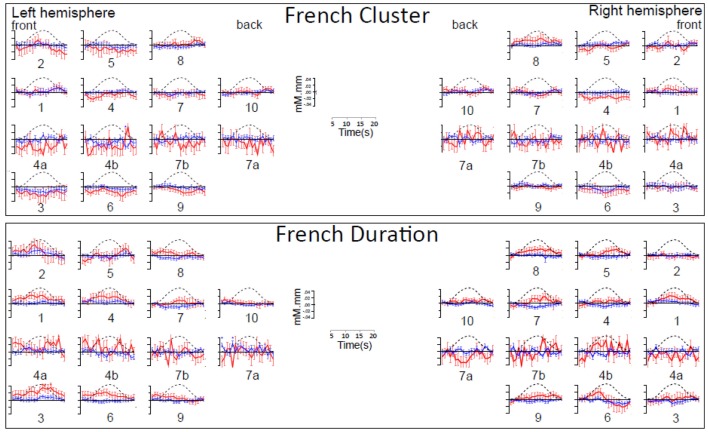
**Time course of hemoglobin responses by condition in French infants (*N* = 20)**. The top panel shows the time courses of oxy-Hb (red) and deoxy-Hb (blue) in the 14 left and 14 right channels recorded in the French infants during Cluster blocks. The bottom panel shows the same for Duration blocks.

**Figure A3:**
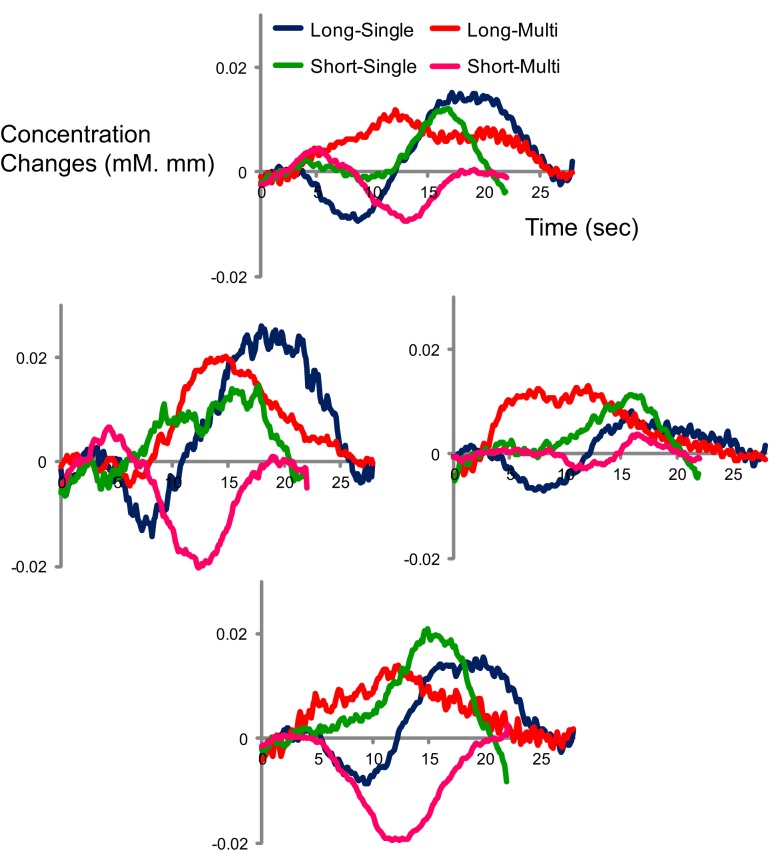
**Time course of oxy-Hb responses by condition in Japanese adults (*N* = 8) in four channels (CH4, 5, 6 and 7 in Figure 1) which correspond to vicinity of auditory area on the left side**.

## Footnotes

^1^A reviewer suggested that analyses methods could also play a role, in that simple averaging could yield stronger signal-to-noise ratios than the ones we reported. We investigated this possibility in the adult data by also calculating averaged Hb within time windows of 7–17 s after the stimulus onset for the long stimulation and 7–14 s for the short one. Although we had similar results to those from GLM [duration *F*(1,31) = 4.93, *p* = 0.061; token variability *F*(1,31) = 18.90, *p* = 0.003], the *F*-values were not much larger using averaging.
